# Reported Symptoms and Associated Factors of Carpal Tunnel Syndrome in Qassim Region: A Cross-Sectional Study

**DOI:** 10.7759/cureus.49385

**Published:** 2023-11-25

**Authors:** Linah S Alduraibi, Rana I Alsamani, Jana M Alfayyadh, Samer A Almuqairsha

**Affiliations:** 1 Internal Medicine, Sulaiman Al-Rajhi University, Al-Bukayriyah, SAU; 2 Internal Medicine, Qassim University, Qassim, SAU; 3 Internal Medicine, Unaizah College of Medicine and Medical Sciences (UCM) at Qassim University, Unaizah, SAU

**Keywords:** prevalence., symptoms, risk factor, bctq, carpal tunnel syndrome

## Abstract

Background

Carpal tunnel syndrome (CTS) is the most common entrapment neuropathy, occurring through compression of the median nerve as it passes under the transverse carpal ligament. Symptoms include nocturnal pain and paresthesias confined to the thumb, middle, and index fingers.

Objective

The purpose of this study is to determine the prevalence of CTS symptoms and the associated risk factors in Qassim Province, Kingdom of Saudi Arabia.

Materials and methods

A total of 314 participants were recruited from Qassim Province using an online survey, which included demographic questions and the Boston Carpal Tunnel Questionnaire (BCTQ). The association between categorical variables was assessed using the Chi-square test or Fisher’s exact test, as appropriate. Additionally, an independent t-test was performed to compare quantitative variables. A p-value of less than 0.05 was considered statistically significant.

Result

The prevalence of CTS in the Qassim population was found to be 19.7%. The most significant risk factors for CTS were age, chronic illnesses, and employment status (p < 0.001). The most frequently reported symptoms included daytime pain and weakness (82.3%), while obesity emerged as the most prevalent chronic disease (21%). The mean symptom severity score (SSS) was significantly higher for individuals who were awoken from pain (t = -5.89; p < 0.001) and for those who experienced awakening due to numbness and tingling (t = -5.59; p < 0.001).

Conclusion

According to our sampled cohort, 19.7% of individuals had symptoms of CTS etiology. Multiple risk factors for CTS were identified. Notably, the development of CTS symptoms was significantly associated with individuals aged 50 and older, employment status, and the presence of related chronic diseases.

## Introduction

Carpal tunnel syndrome (CTS) is the most common entrapment neuropathy. The median nerve at the level of the wrist joint is compressed, and this results in a reduction in the nerve's ability to operate at that location. CTS can manifest in either acute or chronic forms, with the chronic form being significantly more common [1]. The typical symptoms of CTS include pain and paresthesia in the areas served by the median nerve, which encompasses the palmar aspect of the thumb, index, middle fingers, and the radial side of the ring finger [2]. Paresthesia and pain typically dominate early in the course of CTS because sensory fibers are more prone to compression than motor fibers. More severe cases result in motor fiber damage, which weakens the thumb's abduction and opposition. The disappearance of pain may indicate irreversible sensory loss [2]. Generally, the causes of CTS can be divided into occupational and non-occupational categories. Occupational causes often involve jobs that require repetitive and awkward wrist movements for periods less than 30 seconds or activities where such movements constitute more than 50% of the work time. Non-occupational causes may include tumors, ganglion cysts, anatomical anomalies, and trauma [3]. The Boston Carpal Tunnel Syndrome Questionnaire (BCTQ), established by Levine DW et al., was utilized in our study [4]. The BCTQ is known for its good test-retest reliability and validity [5]. It comprises two sections. The first, known as the Symptom Severity Scale (SSS), evaluates the severity of symptoms through eleven questions. The second section, called the Function Status Scale (FSS), focuses on functional aspects and contains eight questions. Each section has scores ranging from 1 to 5, where 1 signifies asymptomatic, 2 mild, 3 moderate, 4 severe, and 5 very severe symptoms [4]. A study on the demographic pattern of CTS in Western Saudi Arabia showed that the mean age was 45.5 years in women and 48.5 years in men. It was reported that the highest age-related gender distribution was among the 45-54 year age group for both males (34.8%) and females (33.9%) [6]. Another study focusing on the awareness of carpal tunnel syndrome among the adult population of Al Majmaah city reported that 14% of participants had CTS [7]. Given the lack of similar research in the Qassim province, the purpose of our study is to determine the prevalence of CTS symptoms and associated risk factors in Qassim province, Kingdom of Saudi Arabia.

## Materials and methods

A cross-sectional study was conducted in the Qassim Province of Saudi Arabia using a qualitative online survey distributed through social media applications. Data collection occurred during March and April 2023, with a total of 314 participants included in the sample. Based on the prevalence of CTS in the Hail region, which was 24.1%, the minimum estimated sample size required was 282 participants, accounting for a 5% margin of error and a 95% confidence interval. The inclusion criteria specified residents of Al-Qassim Province aged 14 years and older from both genders. The study excluded individuals under the age of 14 and those residing outside of Al-Qassim Province.

Data collection methods

All participants were assessed for CTS using a validated and standardized questionnaire known as the BCTQ. The BCTQ comprises two parts: the first part assesses and grades the severity of symptoms, known as the SSS, and the second part, addressing function, is referred to as the FSS. The BCTQ was available in both Arabic and English for the participants. Additionally, we included questions related to demographic information, medical history, symptom location, and other CTS-related queries. We obtained the complete questionnaire with permission from the published study by Altraifi M et al. [8].

Data management and analysis plan

The data was analyzed using IBM SPSS version 29. For qualitative variables, descriptive analyses such as frequency and percentage were applied. Using quartiles, the overall SSS and FSS scores for susceptibility to having CTS symptoms were divided into three categories: mild, moderate, and severe. Scores below the 50th percentile were considered mild, those within the 50th to 75th percentile moderate, and those above the 75th percentile severe. We then assessed the prevalence by focusing on participants with severe symptoms, which fell in the 4th quartile (above the 75th percentile) of the FSS, using a one-sample proportions test. The Chi-square test or Fisher's exact test has been used to discover associations between categorical variables. The independent t-test was used to make a comparison between the qualitative variables. Furthermore, a p-value of less than 0.05 was considered to indicate statistical significance.

Ethical considerations

The study was approved by the Qassim Ethics Research Committee (QERC). In this study, participation was fully voluntary, with the intention of maintaining the confidentiality of all participants' information.

## Results

A total of 314 participants completed the survey in this study. The majority were female, with 277 participants (88.2%), while only 37 participants (11.8%) were male. The 20 to 29 age group had the highest number of participants, accounting for 161 (51.3%) of the total. In terms of BMI, more than half of the participants, 164 (52.2%), had a normal BMI, followed by those who were overweight (72; 22.9%) and obese (50; 15.9%). Additionally, a significant portion of the participants, 266 (72%), were not working. An exhaustive description of the participants' demographic data is provided in Table 1.

**Table 1:** Baseline sociodemographic data for study participants (n=314). ^a^ BMI: Body mass index.

Variable	n (%)
Gender	
Male	37 (11.8%)
Female	277 (88.2%)
Age group	
14-19 years	60 (19.1%)
20-29 years	161 (51.3%)
30-39 years	33 (10.5%)
40-49 years	43 (13.7%)
≥50 years	17 (5.4%)
Nationality	
Saudi	276 (87.9%)
Non-Saudi	38 (12.1%)
City	
Qassim city	298 (94.9%)
Rural side	16 (5.1%)
BMI level^ a^	
Underweight	28 (8.9%)
Normal	164 (52.2%)
Overweight	72 (22.9%)
Obese	50 (15.9%)
Educational level	
Illiterate	1 (0.3%)
Elementary	1 (0.3%)
Intermediate	12 (3.8%)
High school	104 (33.1%)
Higher education	196 (62.4%)
Occupation	
Employed	88 (28%)
Unemployed	226 (72%)

Furthermore, the findings showed a significant association with age in which people >50 years, followed by 40-49 and 20-29 years, were associated with CTS (9; 52.9%, 15; 34.9%, and 30; 18.6%, respectively; p < 0.001). Additionally, having chronic conditions increases the likelihood of complaining of CTS symptoms compared to not having chronic diseases (32; 45.1% and 30; 12.3%, respectively; p < 0.001), and being employed rather than non-employed also had a significant association (26; 29.5% and 36; 15.9%, respectively; p = 0.006). Despite that, there is no significant association with gender or BMI level (p > 0.05) (Table 2).

**Table 2:** Associated factors with prevalence of CTS (n = 314). ^a^ BMI: Body mass index. CTS: Carpal tunnel syndrome. * The statistical significance level is at the 0.05 level.

Characteristics		N	CTS		P-value
			No (n=252, 80.3%)	Yes (n=62, 19.7%)	
Gender	Male	37	31 (83.8%)	6 (16.2%)	0.566
	Female	277	221 (79.8%)	56 (20.2%)	
Age	13-19	60	55 (91.7%)	5 (8.3%)	0.001^*^
	20-29	161	131 (81.4%)	30 (18.6%)	
	30-39	33	30 (90.9%)	3 (9.1%)	
	40-49	43	28 (65.1%)	15 (34.9%)	
	≥50 years	17	8 (47.1%)	9 (52.9%)	
BMI level^ a^	Underweight	28	23 (82.1%)	5 (17.9%)	0.145
	Normal	164	139 (84.8%)	25 (15.2%)	
	Overweight	72	53 (73.6%)	19 (26.4%)	
	Obese	50	37 (74.0%)	13 (26.0%)	
Chronic disease	No	243	213 (87.7%)	30 (12.3%)	0.001^*^
	Yes	71	39 (54.9%)	32 (45.1%)	
Occupation	Employed	88	62 (70.5%)	26 (29.5%)	0.006^*^
	Unemployed	226	190 (84.1%)	36 (15.9%)	

A history of a chronic disease was recorded by 32 of the CTS cases, with obesity (13; 21%), diabetes (12; 19.4%), and hypertension (10; 16.1%) being the most prevalent conditions (Table 3).

**Table 3:** CTS cases with a positive history of a chronic disease (n=32). CTS: Carpal tunnel syndrome.

Chronic diseases	n (%)
Diabetes	12 (19.4%)
Hypertension	10 (16.1%)
Rheumatoid arthritis	8 (12.9%)
Obesity	13 (21%)
Hypothyroidism	8 (12.9%)
Wrist fracture	1 (1.6%)
Other	4 (6.5%)

**Figure 1:**
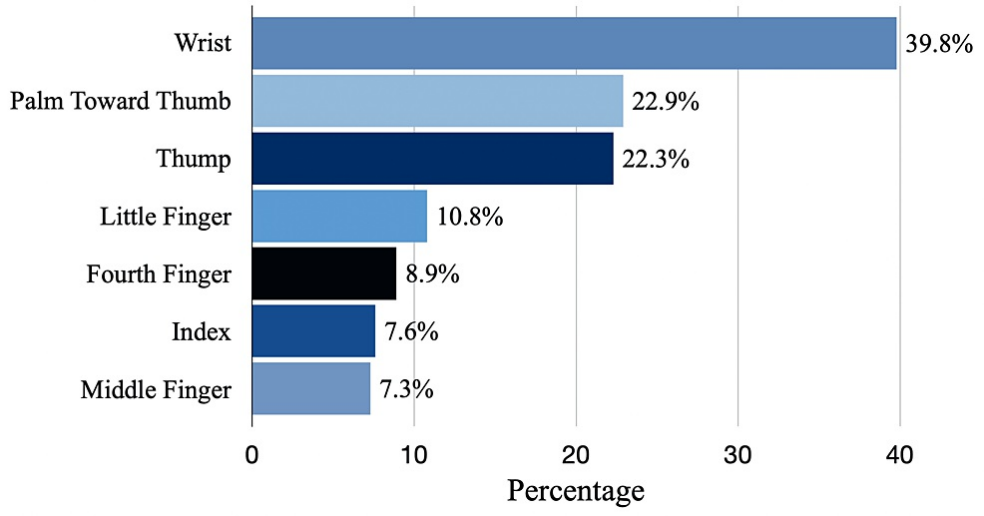
Site of symptoms as reported.

The most frequently reported site of symptoms in CTS cases was the wrist, followed by the palm and thumb (39.8%, 22.9%, and 22.3, respectively), with the middle finger being less frequently recorded (7.3%) (Figure 1).

In CTS cases assessed using the SSS, the following symptoms were commonly reported: pain during the day and weakness in 51 cases (82.3%), pain during the night in 48 cases (77.4%), and numbness and tingling during the night in 46 cases (74.2%). Additional symptoms included muscular atrophy in 7 cases (11.3%) and pain extending to the forearm, reported in 40 cases (64.5%). Furthermore, the majority of cases experienced symptoms in the right hand (29; 46.8%), followed by bilateral symptoms (21; 33.9%) and symptoms in the left hand (12; 19.4%). More detailed information can be found in Table 4.

**Table 4:** Reported symptoms using the symptom severity scale (SSS) in CTS cases (n=62). CTS: Carpal tunnel syndrome.

Variable	n (%)
Pain during daytime	
No	11 (17.7%)
Yes	51 (82.3%)
Numbness during daytime	
No	20 (32.3%)
Yes	42 (67.7%)
Weakness	
No	11 (17.7%)
Yes	51 (82.3%)
Tingling sensation during daytime	
No	21 (33.9%)
Yes	42 (66.1%)
Difficulty grasping	
No	32 (51.6%)
Yes	30 (48.4%)
Pain during night	
No	14 (22.6%)
Yes	48 (77.4%)
Numbness and tingling during the night	
No	16 (25.8%)
Yes	46 (74.2%)
Awoken due to pain at night	
No	29 (46.8%)
Yes	33 (53.2%)
Awoken due to numbness and tingling during the night	
No	27 (43.5%)
Yes	35 (56.5%)

Independent t-tests revealed a statistically significant difference in the mean SSS score between participants who experienced awakening due to pain and those who did not (t = -5.89; p < 0.001). Similarly, there was a significant difference in the mean SSS score between participants who experienced awakening due to numbness and tingling and those who did not (t = -5.59; p < 0.001). Out of the total 314 participants in our study, 166 (52.9%) had mild symptoms, 78 (24.8%) had moderate symptoms, and 70 (22.3%) had severe symptoms on the SSS.

## Discussion

A number of research studies have discussed the prevalence of CTS in various populations. The population prevalence of CTS in this study was 19.7%. In a similar study conducted in the Hail region, the prevalence of symptoms of CTS was 24.1% [8]. In another study conducted in Al Majmaah city, the prevalence of symptoms of CTS was 14% [7]. Given the broad prevalence of warning signs, a study among the general population of Riyadh, Saudi Arabia, revealed a prevalence of 50% for CTS symptoms [9]. In Kuwait, a study conducted among office workers revealed that 18.7% have CTS symptoms [10]. Unlike other studies, this study shows that gender is not significantly associated with the development of CTS symptoms. In contrast, other studies show a significant association and mention that females tend to be more affected than males [8,9,11,12]. CTS symptoms were approximately more common in people who were overweight and obese than in those who were not, although our research did not find any significance in this. According to numerous studies, BMI is a significant risk factor for the development of CTS since people with a BMI of 30 or above are more likely to be affected [12-14]. In our study, the age range of 50 and older was a significant predictor of the emergence of CTS symptoms. The older age group (41-60 years) was also demonstrated to be a significant risk factor in a Brazilian study [12]. Studies conducted in Hail and Riyadh showed that younger people reported symptoms more commonly than others [8,9]. Few other studies reported no association between a specific age group and CTS development [11,15]. Also, our study shows that chronic conditions and employment are significantly associated with the development of CTS symptoms, as CTS symptoms are more prevalent in those with chronic conditions and office workers, which are also mentioned in Hail and Kuwait studies. Additionally, the wrist, palm, and thumb were the most commonly reported sites of symptoms in CTS cases (39.8%, 22.9%, and 22.3%), with the middle finger being less frequently reported (7.3%). However, a study conducted in Hail reported the location of symptoms as follows: the palm of the hand (47.4%), wrist (47.3%), and thumb (36.1%) [8]. Furthermore, muscular atrophy (7; 11.3%) and pain that occasionally radiates to the forearm (40; 64%) were also reported. The majority of cases (29; 46.8%) present with symptoms in the right hand, followed by bilateral (21; 33.9%) and left hands (12; 19.4%).

Moreover, 32 of the CTS cases had a history of chronic diseases, with the most common conditions being obesity (21%), diabetes (19.4%), hypertension (16.1%), rheumatoid arthritis (8.9%), hypothyroidism (8.9%), and less frequently, wrist fracture (1.6%). This distribution is similar to the Hail study, which reported that 32 (22.7%) people had obesity, 12.5% had diabetes, 12.5% had hypertension, and 13.2% had rheumatoid arthritis [8]. In our study, we found that out of the 314 participants, 166 (52.9%) had mild symptoms, 78 (24.8%) had moderate symptoms, and 70 (22.3%) had severe symptoms on the SSS. This study has a number of strengths. It provides updated information about common symptoms using a validated standardized questionnaire called the BCTQ, which consists of two parts: the SSS, a tool for assessing and grading the severity of symptoms, and the FSS, which deals with function. In addition, this study is not limited to demographic characteristics of gender and age. Other significant risk factors for CTS, particularly diabetes mellitus, obesity, and hypertension, which are common diseases, were addressed in this study.

## Conclusions

Our study showed that the prevalence of CTS symptoms in the Qassim region is 19.7%. We identified multiple risk factors for developing CTS. Ages 50 and up were significantly associated with the development of CTS symptoms, as were individuals with associated chronic conditions and those who were employed. We recommend further research using nerve conduction tests and clinical evaluation.
